# Cordycepin Inhibits Enterovirus A71 Replication and Protects Host Cell from Virus-Induced Cytotoxicity through Adenosine Action Pathway

**DOI:** 10.3390/v16030352

**Published:** 2024-02-24

**Authors:** Yi-Ping Lee, Chun-Keung Yu, Tak-Wah Wong, Li-Ching Chen, Bu-Miin Huang

**Affiliations:** 1 Department of Cell Biology and Anatomy, College of Medicine, National Cheng Kung University, Tainan 70101, Taiwan; yipinglee@hotmail.com; 2 Department of Microbiology and Immunology, College of Medicine, National Cheng Kung University, Tainan 70101, Taiwan; dckyu@mail.ncku.edu.tw; 3 Institute of Basic Medical Sciences, College of Medicine, National Cheng Kung University, Tainan 70101, Taiwan; 4 Center of Infectious Disease and Signaling Research, College of Medicine, National Cheng Kung University, Tainan 70101, Taiwan; 5 Department of Dermatology, National Cheng Kung University Hospital, College of Medicine, National Cheng Kung University, Tainan 70101, Taiwan; twwong@mail.ncku.edu.tw; 6 Department of Biochemistry and Molecular Biology, College of Medicine, National Cheng Kung University, Tainan 70101, Taiwan; 7 Center of Applied Nanomedicine, National Cheng Kung University, Tainan 70101, Taiwan; 8 Department of Biological Science & Technology, China Medical University, Taichung 406040, Taiwan; 9 Department of Medical Research, China Medical University Hospital, China Medical University, Taichung 40402, Taiwan

**Keywords:** cordycepin, enterovirus A71, antiviral activity, adenosine pathway, traditional herbal medicine, infectious disease

## Abstract

Enterovirus A71 (EV-A71) infection typically causes mild illnesses, such as hand-foot-and-mouth disease (HFMD), but occasionally leads to severe or fatal neurological complications in infants and young children. Currently, there is no specific antiviral treatment available for EV-A71 infection. Thus, the development of an effective anti-EV-A71 drug is required urgently. Cordycepin, a major bioactive compound found in *Cordyceps* fungus, has been reported to possess antiviral activity. However, its specific activity against EV-A71 is unknown. In this study, the potency and role of cordycepin treatment on EV-A71 infection were investigated. Results demonstrated that cordycepin treatment significantly reduced the viral load and viral ribonucleic acid (RNA) level in EV-A71-infected Vero cells. In addition, EV-A71-mediated cytotoxicity was significantly inhibited in the presence of cordycepin in a dose-dependent manner. The protective effect can also be extended to Caco-2 intestinal cells, as evidenced by the higher median tissue culture infectious dose (TCID_50_) values in the cordycepin-treated groups. Furthermore, cordycepin inhibited EV-A71 replication by acting on the adenosine pathway at the post-infection stage. Taken together, our findings reveal that cordycepin could be a potential antiviral candidate for the treatment of EV-A71 infection.

## 1. Introduction

Enterovirus A71 (EV-A71) infection is an emerging infectious disease that has become a serious threat to public health around the world in recent years, especially in the Asia-Pacific region [1]. EV-A71 belongs taxonomically to the genus Enterovirus in the family Picornaviridae [2,3]. The common infectious age groups are infants and young children, and it is transmitted through the fecal–oral route [4,5]. The genome of EV-A71 consists of a single-stranded positive polar ribonucleic acid (RNA) of approximately 7.4 kb in length. Its open reading frame can be translated into 11 functional viral proteins, including 4 structure proteins (VP1, VP2, VP3, and VP4) and 7 non-structure proteins (2A, 2B, 2C, 3A, 3B, 3C, and 3D) [6]. After EV-A71 infects the host, it first invades gastrointestinal cells and utilizes them as the primary replication site [7]. Infection is initiated when the virus binds to a specific receptor on the cell surface, such as scavenger receptor class B, member 2, and then enters the cytoplasm of the target cell using receptor-mediated endocytosis [2,8]. When the virus enters the cell, the capsid will disintegrate due to the acidic environment of the vesicle, and then release the genome into the cytoplasm. Viral RNA can be used directly as a template to translate a polyprotein. Then, this polyprotein will be cleaved into individual viral proteins by its own protease activity. Simultaneously, the virus also uses RNA-dependent RNA polymerase to replicate the genome. Then, these newly synthesized viral proteins and RNA genomes are assembled to form a new generation of viral particles. Eventually, the viruses are released from the infected host cells and infect neighboring cells [6]. Potential drug candidates may form blockades to various stages of the EV-A71 life cycle [2]. Once infection is established at the primary replication site, the virus may spread to other tissues and organs, causing different types of complications, such as hand-food-and-mouth disease (HFMD) and herpangina [9]. In addition, due to the neurotropism of EV-A71, it may occasionally invade the central nervous system (CNS), causing severe neurological complications, such as aseptic meningitis, poliomyelitis-like paralysis, brainstem encephalitis, and neurogenic pulmonary edema (PE) [9,10,11].

Clinically, several EV-A71 vaccines have been approved for use in the prevention of infectious diseases, but there are no specific antiviral drugs or therapies that can be used to combat EV-A71 infection. Therefore, the development of an anti-EV-A71 drug is required urgently to stop viral replication, and thus relieve symptoms and prevent the development of severe complications.

With the advancement of science and technology, many strategies are currently used in the screening, design, and development of antiviral drugs, including in silico drug discovery [12], structure-based drug design [13], high-throughput screening [14], and repurposing approved drugs [15]. Through these strategies, different types of drug candidates with therapeutic potential can be obtained, such as nucleoside analogs [16], polymerase inhibitors [17], protease inhibitors [18], monoclonal antibodies [19], RNA interferences [20], and natural compounds from traditional medicine [21]. Thus far, many candidate drugs have been reported to have the potential to treat EV-A71 infectious disease. Type I interferon (IFN) has been reported to have good anti-EV-A71 ability [22,23,24]. It can protect the host from EV-A71 infection by directly inhibiting viral replication or regulating the immune response. Milrinone, a type III phosphodiesterase inhibitor, has been reported to improve the survival rate of patients with EV-A71-related neurological disorders due to its superior ability to regulate inflammation and reduce cytokine storms [25,26]. It has been clinically recommended as a complementary therapy for patients with brainstem encephalitis and PE caused by EV-A71. In addition, some reports suggest that intravenous immune globulin (IVIG) given to patients with HFMD early in the infection can slow the progression of the disease and reduce mortality [27,28,29]. Moreover, antiviral agents such as ribavirin and pleconaril have also shown a positive effect in the treatment of EV-A71 infectious disease to some extent [30]. Traditional herbal medicines (THMs) have a long history of use in the Asia-Pacific region as nutritional supplements or alternative therapies to treat diseases. Some THMs, such as honeysuckle [31], forsythia [32], and *Cordyceps sinensis* [33], have been scientifically proven to have antiviral effects and have been used in traditional medical practices to treat certain viral infections. Furthermore, recent research results also provide strong evidence that some nucleic acid, nucleoside, nucleotide, deoxyribonucleic acid (DNA), and RNA analogs have excellent antiviral effects [34].

**Figure 1:**
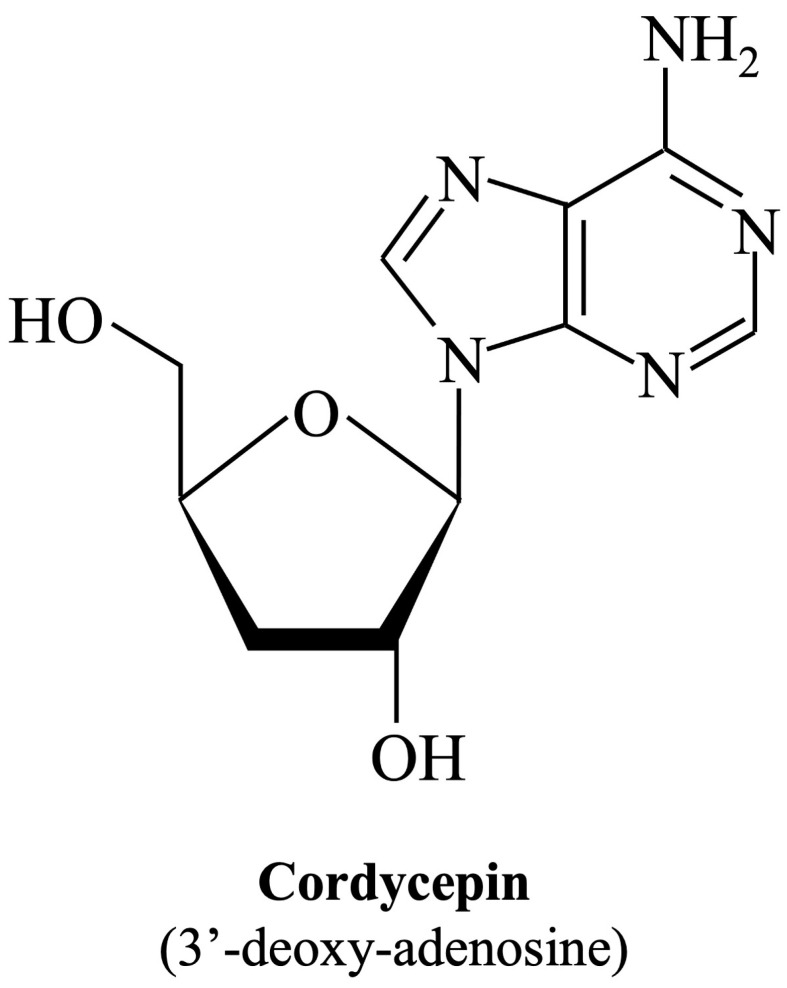
Chemical structure of cordycepin. The chemical structure is shown in the illustration. The International Union of Pure and Applied Chemistry Chemical Nomenclature (IUPAC) name is in brackets.

Cordycepin (IUPAC name: 3′-deoxy-adenosine; Figure 1), an adenosine analog, is the main functional component of *Cordyceps sinensis*, which has been used extensively as an herbal complementary and alternative medicine drug to treat different illnesses based on its multiple pharmacological activities such as immune regulation, apoptosis, or autophagy modulation, as well as cellular oxidative stress induction [35]. The main biological activity of cordycepin is probably mediated by cell surface receptors such as adenosine receptors. After binding to receptors, it affects various types of cells, tissues, and organ systems through intracellular and extracellular signaling pathways [36]. Moreover, cordycepin has been shown to suppress the replication of several viruses, including dengue virus [37], Epstein–Barr virus [38], poliovirus [39], and severe acute respiratory syndrome coronavirus 2 (SARS-CoV-2) [40]. Whether cordycepin also has the ability to combat EV-A71, and if so, through what role it achieves its effect, are mysteries that remain.

Once cordycepin is confirmed to have anti-EV-A71 activity, and has become a candidate drug for the treatment of EV-A71; its clinical significance and future applications may focus on using cordycepin in the treatment of mild EV-A71 infections such as HFMD or herpangina to reduce symptoms, shorten the clinical course, and reduce the risk of serious complications. On the other hand, a good effect of cordycepin treatment could be expected from a prophylactic course of treatment, as used in traditional Chinese medicine to combat EV-A71 infection. In addition, the combination treatment of cordycepin and other drug candidates with complementary mechanisms may improve the therapeutic potential. Epidemiologically, outbreak areas of enterovirus infection often contain a prevalence of different enteroviruses and human parechoviruses [5,41]. Although different enteroviruses and human parechoiruses may cause different clinical manifestations in the host [5], based on the fact that these viruses share similar infection and replication strategies [5], the anti-EV-A71 effect of cordycepin should theoretically be broadly applicable to other enteroviruses and human parechoviruses.

In this study, we demonstrated that the cordycepin treatment could effectively inhibit EV-A71 replication, and further protect host cells from virus-induced cytotoxicity through an adenosine action pathway at the post-infection stage.

## 2. Materials and Methods

### 2.1. Cells and Viruses

Rhabdomyosarcoma (RD), Vero, and Caco-2 cells were maintained in Dulbecco’s modified Eagle’s medium (DMEM) containing 20% (for Caco-2 cells) or 10% (for other cells) fetal bovine serum (FBS) with 2 mM L-glutamine, 100 IU/mL penicillin, and 100 μg/mL streptomycin. The cells were cultured in a humidified incubator containing 5% CO_2_ at 37 °C. EV-A71/MP4 (GenBank accession no. JN544419) and EV-A71/Tainan/4643/98 (GenBank accession no. AF304458) viruses were propagated in RD cells. Viral titers were quantitated using plaque assays. Working stocks of EV-A71/MP4 and EV-A71/Tainan/4643/98 contained 1.0 × 10^8^ and 1.2 × 10^8^ plaque-forming units (pfu)/mL, respectively.

### 2.2. Chemicals

Cordycepin and adenosine were purchased from Sigma-Aldrich. The purities of the cordycepin and adenosine, as identified by high-performance liquid chromatography, were more than 98% and 99%, respectively. The cordycepin and adenosine were dissolved in dimethyl sulfoxide (Sigma-Aldrich, St. Louis, MI, USA) to a final concentration of 80 mM as a stock solution.

### 2.3. Virus Infection

**Figure 2:**
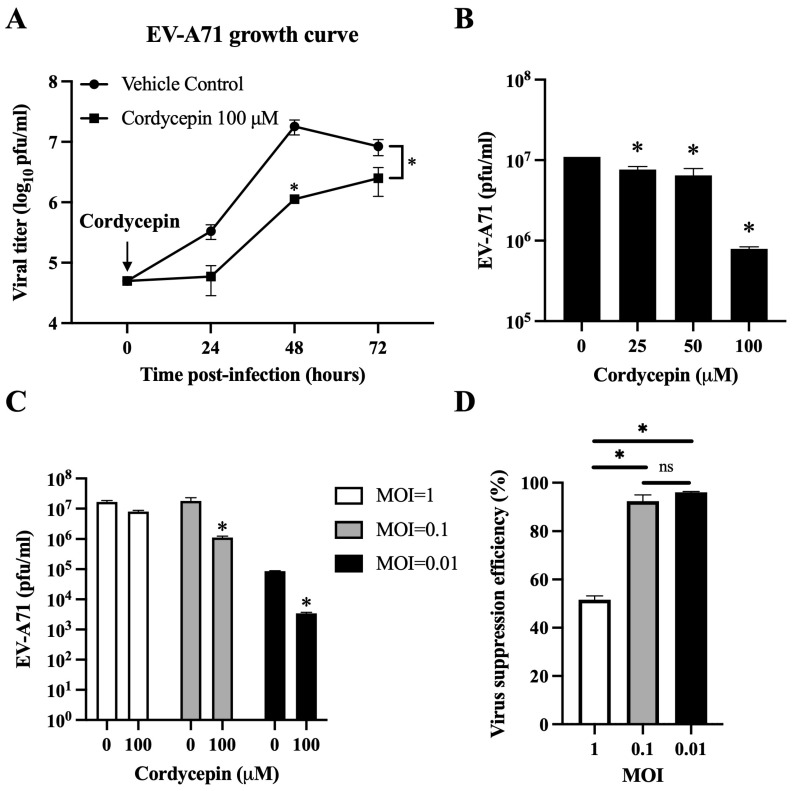
Cordycepin reduces the viral load of EV-A71 in Vero cells. EV-A71/MP4 viruses were added to Vero cell cultures at an MOI of 0.01, 0.1, or 1 in the absence or presence of 25, 50, or 100 μM cordycepin for 24, 48, or 72 h. Then, cultures (cells with supernatants) were harvested for viral titer detection using plaque assays. (**A**) One-step growth curves are shown. (**B**) The inhibitory effect on the viral load of different cordycepin doses at 48 h and (**C**) the inhibitory effect on the viral load of 100 μM cordycepin at different MOIs at 48 h were compared. (**D**) The viral suppression efficiency percentages were calculated from the viral titers of 100 μM cordycepin-treated groups relative to the vehicle control group under different MOIs at 48 h. Results are presented as mean ± SEM of three independent experiments. * *p* < 0.05 represents significant statistical difference. ns, no significant difference.

**Figure 3:**
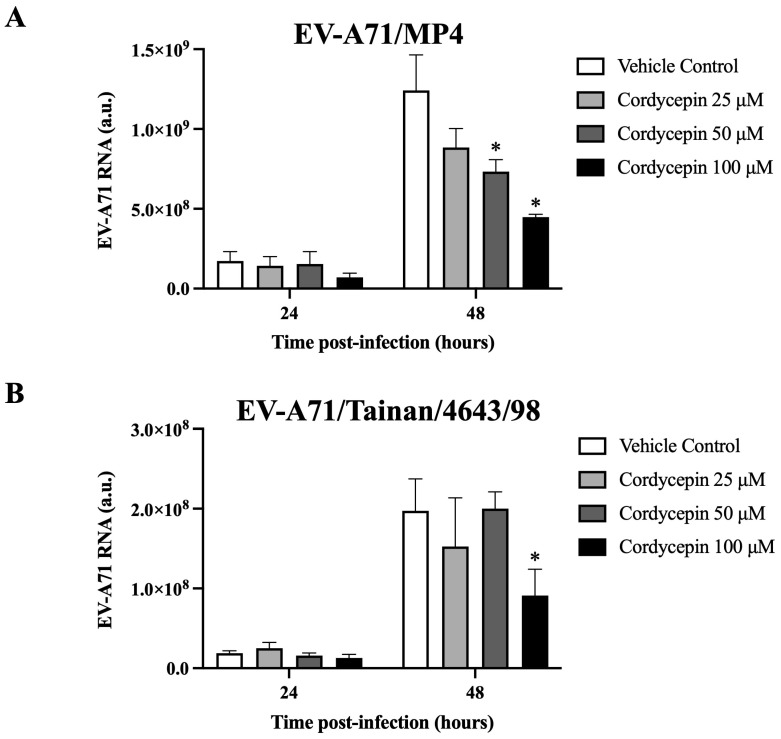
EV-A71 RNA level decreased in virus-infected Vero cells in the presence of cordycepin. (**A**) EV-A71/MP4 or (**B**) EV-A71/Tainan/4643/98 viruses were added to Vero cell cultures at an MOI of 0.1 in the absence or presence of 25, 50, or 100 μM cordycepin for 24 and 48 h. Then, cultures (cells with supernatants) were harvested for RNA isolation, followed by conversion to cDNA. Viral RNA levels were quantified by real-time RT-PCR with EV-A71-specific primer pairs, and normalized by the mRNA levels of GAPDH. Data are presented as mean ± SEM of three independent experiments. * *p* < 0.05 represents significant statistical difference compared to the vehicle control group. a.u., arbitrary unit.

**Figure 4:**
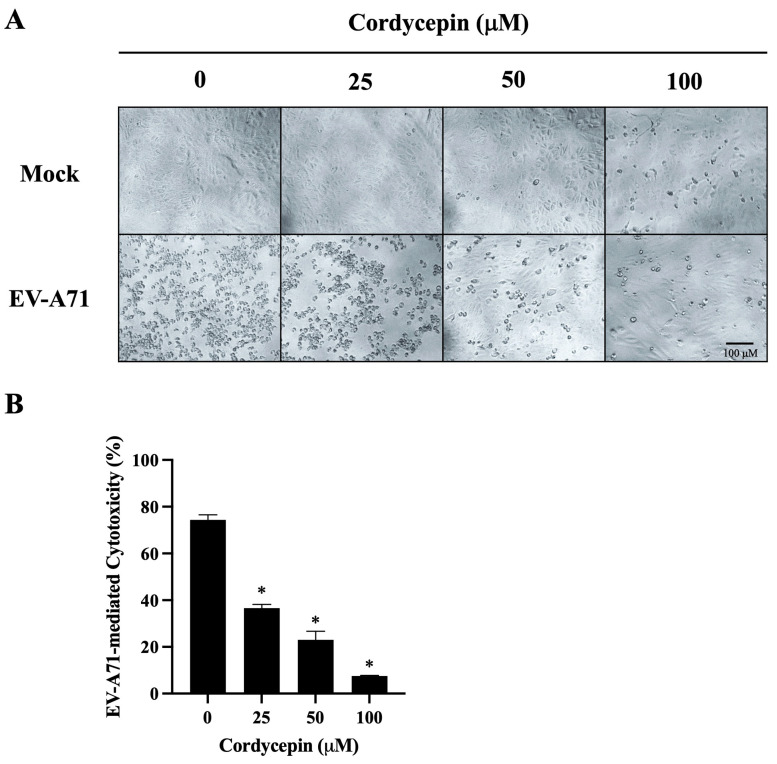
EV-A71-mediated cytotoxicity in Vero cells is decreased in the presence of cordycepin. EV-A71/MP4 viruses were added to Vero cell cultures at an MOI of 0.1 in the absence or presence of 25, 50, or 100 μM cordycepin for 96 h. (**A**) The morphology of cells was examined using a light microscope with 100x magnification, and images were captured by digital camera. (**B**) Cell viability was examined using the CCK-8 viability test. The percentages of EV-A71-mediated cytotoxicity were calculated with the formula mentioned in the Materials and Methods section. Data are presented as mean ± SEM of three independent experiments. * *p* < 0.05 represents significant statistical difference compared to the untreated control group.

In the experiments shown in Figure 2, Figure 3 and Figure 4, EV-A71/MP4 or EV-A71/Tainan/4643/98 were added directly to Vero or Caco-2 cell cultures at a multiplicity of infection (MOI) of 0.01, 0.1, or 1 for 24, 48, 72, or 96 h. In the cytopathic effect (CPE) protection assay, serially diluted virus suspensions were used to infect cells instead of fixed MOI infectious conditions for 72 h. During the addition assay, immunofluorescence assay, and adenosine competition assay, EV-A71/MP4 viruses were added to cell cultures for 1-h adsorption, and then unbound viruses were removed by washing twice with phosphate-buffered saline (PBS).

### 2.4. Plaque Assay

RD cells (3 × 10^5^ cells/well) were seeded in 24-well plates. After attachment, the medium was removed, and 100 μL serially diluted viral suspensions were added for 1-h adsorption. Then, the viral suspension was replaced with 800 μL DMEM containing 2% FBS and 0.5% methylcellulose. After incubation for 72 h, the medium was removed and cell monolayers were stained with crystal violet solution (0.5% crystal violet, 10% formalin, and 10% ethanol) for 1 h. Then, the plate was washed with water and dried. The number of plaques was counted, and the viral titer was calculated using the following formula [42]:

$$Viral\;titer\;(pfu/mL)=\frac{verage\;number\;of\;plaques}{Dilution\times Volume\;of\;diluted\;sample\;added\;to\;the\;well\;(\mathrm{mL})}$$

### 2.5. RNA Extraction and Real-Time Reverse Transcription-Polymerase Chain Reaction (RT-PCR)

Culture (cells with supernatants) was harvested and subjected to three freeze–thaw cycles for intracellular viral particle release, and then homogenized in TRIzol LS reagent (Invitrogen, Waltham, MA, USA). The sample was mixed with chloroform (1/6 volume) and centrifuged for 15 min at 12,000× *g* at 4 °C after incubation at room temperature (RT) for 3 min. The aqueous phase containing the RNA was transferred to a new tube and mixed with 70% ethanol (1/4 volume). High-purity RNA was extracted using the RNeasy Mini Kit (Qiagen, Hilden, Germany) according to the manufacturer’s instructions. A mass of 5 μg RNA was used to synthesize complementary DNA (cDNA) with the SuperScipt IV First-strand cDNA Synthesis Kit (Invitrogen) with oligo d(T)_20_ primer (Invitrogen) in a reaction volume of 20 μL. Levels of simian glyceraldehyde 3-phosphate dehydrogenase (GADPH) messenger RNA (mRNA), EV-A71/MP4 viral RNA, and EV-A71/Tainan/4643/98 viral RNA were detected using specific primer pairs (Table 1) with Fast SYBR Green Master Mix (Applied Biosystems, Waltham, USA) and measured using the StepOnePlus real-time PCR system (Applied Biosystems). The relative viral RNA quantity was calculated with the 2^−ΔΔCT^ threshold cycle (CT) method and normalized to the quantity of GAPDH. Calculation of the arbitrary units (a.u.) was described previously [43].

### 2.6. Cell Viability Test

Vero cells (5 × 10^3^ cells/well) were seeded in a 96-well plate. After 70–80% confluence, the cells were infected without or with EV-A71/MP4 at an MOI of 0.1 in the absence or presence of 25, 50, or 100 μM cordycepin in a final volume of 100 μL for 96 h. The morphology of the cells was examined using a light microscope, and images were captured. A volume of 10 μL of cell counting kit (CCK)-8 solution (Sigma-Aldrich) was added to each well and incubated at 37 °C for 3 h. After incubation, optical density (O.D.) values were determined under absorbance at 450 nm by an enzyme-linked immunosorbent assay (ELISA) reader. The percentages of EV-A71-mediated cytotoxicity were calculated with the following formula (*A*: Absorbance):

$$EV-A71-mediated\;cytotoxicity\left(\%\right)\;=\frac{A_{Mock\left(individual\;dose\right)}-A_{EV-A71(individual\;dose)}}{A_{Mock(vehicle\;control)}}\times 100\%$$

### 2.7. CPE Protection Assay

Vero or Caco-2 cells (5 × 10^3^ cells/well) were seeded in a 96-well plate and cultured for 18 h. The culture medium was removed, and cells were infected with or without serially diluted EV-A71/MP4 or EV-A71/Tainan/4643/98 viral suspensions in the absence or presence of 25, 50, or 100 μM cordycepin in a final volume of 100 μL. CPE was examined using a light microscope after incubation for 72 h. The infection rate was calculated using the following formula:

$$\begin{array}{l} Infection\;rate\left(\%\right)\\ =\frac{Number\;of\;cumulative\;positive\;units}{Number\;of\;cumulative\;positve\;units+Number\;of\;cumulative\;negative\;units} \end{array}$$

The dilution corresponding to the 50% endpoint lay between two dilutions; the proportionate distance (PD) between these two dilutions was calculated with the following formula:

$$PD=\frac{\%positive\;above\;50\%-50\%}{\%positive\;above\;50\%-\%positive\;below\;50\%}$$

Then, the logTCID_50_ value was determined using the Reed–Muench method, as follows [44]:

$$\log{\mathrm{TCID}}_{50}=\log\left(Dilution\;with>50\%\;positive\right)+PD\times \left[-\log\left(Dilution\;factor\right)\right]$$

### 2.8. Time of Addition Assay

A 100 μM cordycepin concentrate was added to Vero cell cultures (overnight culture of 1 × 10^6^ cells/well in a 6-well plate) or viral suspensions (1.0 × 10^5^ pfu/mL) in four different experimental settings to test how cordycepin interfered with the infection of EV-A71 on cells. To test whether cordycepin directly affected EV-A71 infectivity, viral suspensions of EV-A71/MP4 were pre-mixed with cordycepin for 30 min at 37 °C, then added to Vero cells, and removed after 1-h virus adsorption (setting 1: pre-incubation with virus). To test the possibility that cordycepin affected the host cells before viral infection, cordycepin was incubated with the cells for 30 min. Then, the cells were infected with EV-A71/MP4 at an MOI of 0.1 after the medium was removed (setting 2: pre-incubation with cell). To test whether cordycepin affected receptor binding or the viral entry step, cordycepin and EV-A71/MP4 were simultaneously added to the cells and removed after virus adsorption (setting 3: co-infection). To test the effect of cordycepin on viral replication after viral entry, cordycepin was added after virus adsorption (setting 4: post-infection). Viral RNA levels of cultures from different settings were quantified as described above.

### 2.9. Immunofluorescence Assay

Vero cells were mock-infected or infected with EV-A71/MP4 at an MOI of 0.1 and treated with or without 100 μM cordycepin after 1-h virus adsorption for 48 h. After incubation, the cells were harvested, dropped to a microscope slide, and fixed with acetone at 4 °C. The slide was blocked with PBS containing 3% bovine serum albumin (BSA) and incubated with an EV-A71 VP1 polyclonal antibody (Invitrogen, PA5-111996, 1:250 dilution). After washing, the slide was subsequently incubated with Alexa Fluor 488 conjugated goat anti-rabbit immunoglobulin G (IgG) (Invitrogen, A-11008, 1:500 dilution). Finally, the slide was sealed by a coverslip with EverBrite mounting medium with 4′,6-diamidino-2-phenylindole (DAPI) (Biotium, Fremont, USA) and visualized under a fluorescence microscope. The fluorescence intensity was analyzed using ImageJ software, version 1.53.

### 2.10. Adenosine Competition Assay

Vero cells were infected by EV-A71/MP4 at an MOI of 0.1, and then treated with or without 100 μM cordycepin and increasing doses of adenosine after 1-h virus adsorption. To assess the effect of adenosine competition on viral genome replication, cultures were harvested for RNA isolation at 48 h post-infection, followed by conversion to cDNA. The viral RNA levels were quantified as described above. To examine the effect of adenosine competition on CPE protection, Vero cells were infected with a serial dilution of EV-A71/MP4 in a 96-well plate, and then treated with or without 100 μM cordycepin and adenosine (0 or 100 μM) after 1-h virus adsorption. After 72 h, CPE was examined and TCID_50_ values were calculated using the Reed and Muench formula described above.

### 2.11. Statistical Analysis

The data are expressed as mean ± standard error of the mean (SEM) of three separate experiments. Statistically significant differences were determined via one-way or two-way analysis of variance (ANOVA) or Student’s *t*-test. The statistical significance was considered as *p* < 0.05 in all experiments.

## 3. Results

### 3.1. Cordycepin Reduces the Viral Load of EV-A71 in Vero Cells

Cordycepin has been shown to inhibit dengue virus replication at a dose of 100 μM in Vero cells without obvious cytotoxicity [37]. Thus, we first used this dosage to test the inhibitory effect of cordycepin on the viral load of EV-A71 at an MOI of 0.1 in Vero cells. The viral load of EV-A71/MP4 in Vero cells was significantly suppressed in the presence of 100 μM cordycepin, as shown by a gentle growth curve in the log phase, and an approximately 16-fold decrease in viral titer at 48 h post-infection as compared to the vehicle control (*p* < 0.05) (Figure 2A). In this time point, cordycepin significantly reduced the EV-A71/MP4 viral titers in Vero cells in a dose-dependent manner (*p* < 0.05, Cordycepin 25/50/100 μM vs. Cordycepin 0 μM) (Figure 2B). Furthermore, 100 μM cordycepin significantly reduced the titers of EV71/MP4 in Vero cells at MOIs of 0.01 and 0.1 (*p* < 0.05), but not an MOI of 1 (*p* > 0.05) at 48 h post-infection (Figure 2C), with suppression efficiencies of 96%, 92%, and 52%, respectively (Figure 2D).

### 3.2. EV-A71 RNA Level Is Decreased in Virus-Infected Vero Cells in the Presence of Cordycepin

We further determined whether cordycepin could interfere with the genomic replication of EV-A71. The results demonstrate that in the presence of 50 μM or 100 μM cordycepin, the RNA level of EV-A71/MP4, a virulent strain for laboratory mice, was significantly and dose-dependently reduced at 48 h post-infection as compared to the nontreated control (*p* < 0.05) (Figure 3A). Similar results were also observed in a clinical isolated strain, EV-A71/Tainan/4643/98 (Figure 3B).

### 3.3. EV-A71-Mediated Cytotoxicity in Vero Cells Is Decreased in the Presence of Cordycepin

We further observed the morphological changes of mock- or EV-A71/MP4-infected Vero cells in the absence or presence of cordycepin at 96 h post-infection. As shown in Figure 4A, mock-infected Vero cells were firmly attached and exhibited the commonly anticipated polygonal-shaped morphology on the cell culture plates, regardless of whether cordycepin was administered or not; very few cells became rounded in the presence of 100 μM cordycepin. Conversely, cells infected with EV-A71/MP4 in the absence of cordycepin became rounded, more detached, with cell membrane blebbing and shrinkage, reflecting the severe CPE of cells caused by infection. The CPE induced by EV-A71 was alleviated with the increase in cordycepin concentration, and only a few cells exhibited a CPE in the presence of 100 μM cordycepin at 96 h post-infection (Figure 4A). In addition, under the mock infectious condition, there was no significant difference in the O.D. values of the cell viability test between the 25, 50, or 100 μM cordycepin-treated group and the nontreated control group (*p* > 0.05) (Figure S1A). This result indicates that the dose of cordycepin that we tested in this study does not induce obvious cytotoxicity to Vero cells. Under the condition of EV-A71 infection, the O.D. values of the 50 and 100 μM cordycepin-treated groups significantly increased by more than 2-fold compared with the nontreated control group (*p* < 0.05; Figure S1B). Markedly, the percentages of EV-A71-mediated cytotoxicity were significantly decreased by the increase in cordycepin concentration in a dose-dependent manner (*p* < 0.05) (Figure 4B).

### 3.4. TCID_50_ Values of EV-A71 in Vero and Caco-2 Cells Are Increased in the Presence of Cordycepin

**Figure 5:**
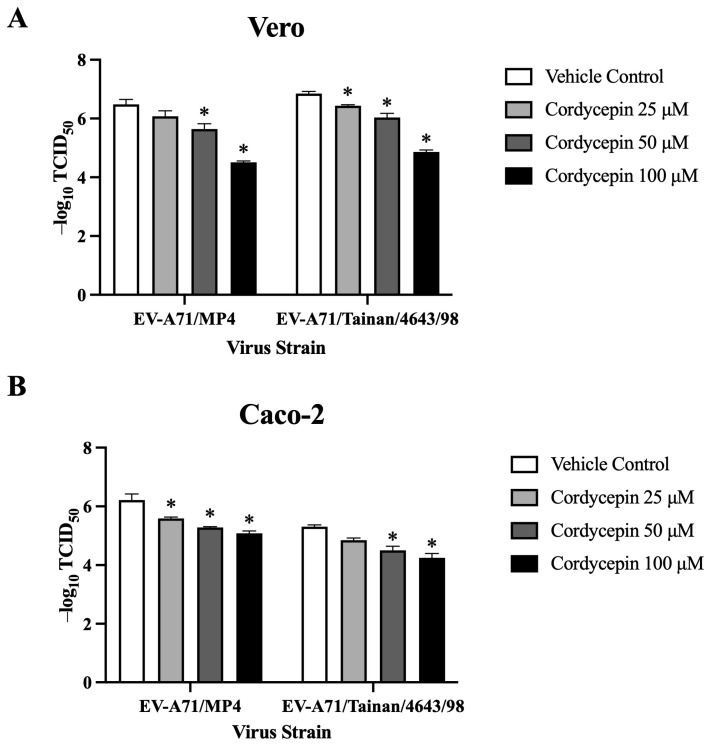
TCID_50_ values of EV-A71 in Vero and Caco-2 cells are increased in the presence of cordycepin. (**A**) Vero and (**B**) Caco-2 cells were seeded and cultured in 96-well plates for 18 h. Then, the cell monolayers were infected with a serial dilution of EV-A71/MP4 or EV-A71/Tainan/4643/98 in the absence or presence of 25, 50, or 100 μM cordycepin for 72 h. The cytopathic effect (CPE) was examined, and TCID_50_ values were calculated using the Reed and Muench formula. Data are presented as mean ± SEM of three independent experiments. * *p* < 0.05 represents significant statistical difference compared to the vehicle control group.

To further characterize the role of cordycepin in protecting host cells from EV-A71-mediated cytotoxicity, we used the CPE protection assay to detect changes in TCID_50_ values of EV-A71-infected cells in the absence or presence of different concentrations of cordycepin. In this part of the experiments, we additionally included Caco-2 intestinal cells to determine whether the protection phenomenon can be extended to different EV-A71 target cells. Similar to the results in Vero cells, 25, 50, and 100 μM cordycepin did not cause significant cytotoxicity to Caco-2 cells (*p* > 0.05, cordycepin 25/50/100 μM vs. cordycepin 0 μM) (Figure S2). Conversely, EV-A71/MP4 and EV-A71/Tainan/4643/98 infections led to a noticeable CPE in Vero, and Caco-2 cells. However, the presence of cordycepin resulted in a reduction in CPE in a dose-dependent manner, as indicated by higher TCID_50_ values compared to the vehicle control (Figure 5). Overall, cordycepin treatment at concentrations greater than or equal to 50 μM could significantly increase the TCID_50_ values of EV-A71/MP4 and EV-A71/Tainan/4643/98 infections in Vero (Figure 5A), and Caco-2 (Figure 5B) cells as compared to the vehicle control (*p* < 0.05).

### 3.5. Cordycepin Inhibits EV-A71 Replication after Cellular Entry

**Figure 6:**
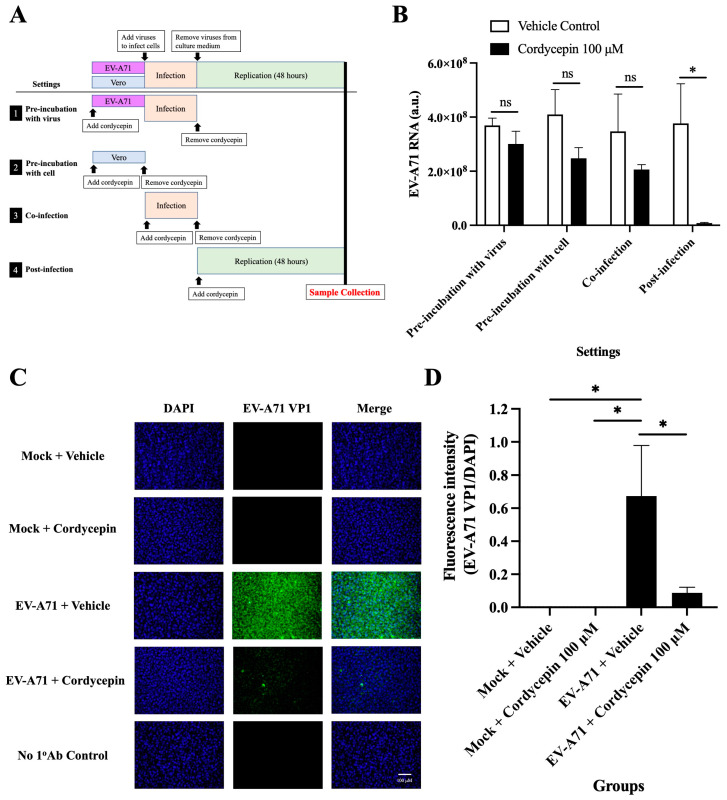
Cordycepin inhibits EV-A71 replication after cellular entry. To test the exact impact stage of the anti-EV-A71 effect of cordycepin, 100 μM cordycepin was administrated to the Vero cell cultures in four different experimental settings, including pre-incubation with virus, pre-incubation with cell, co-infection, and post-infection. (**A**) The period of the cordycepin action is shown in the illustration. (**B**) Then, cultures (cells with supernatants) were harvested for RNA isolation followed by conversion to cDNA. Viral RNA levels were quantified by real-time RT-PCR with EV-A71-specific primer pairs and normalized by the mRNA levels of GAPDH. The inhibitory effects of cordycepin were compared for different addition steps. (**C**) The numbers of EV-A71-infected cells after treatment with 100 μM cordycepin at the post-infection stage were examined at 48 h by immunofluorescence assay. EV-A71 VP1 protein was stained green by a specific antibody with Alexa Fluor 488-conjugated secondary antibody, and nuclei were stained blue with DAPI. (**D**) The fluorescence intensity ratios of EV-A71 VP1 per DAPI were analyzed with ImageJ software. Data are presented as mean ± SEM of three independent experiments. * *p* < 0.05 represents significant statistical difference. ns, no significant difference.

To test the exact action stage of the anti-EV-A71 effect of cordycepin, Vero cells were infected with EV-A71/MP4, and the time of addition assay was performed (Figure 6A). The results showed that treatment with 100 μM cordycepin did not alter the levels of EV-A71 RNA in the “pre-incubation with the virus”, “pre-infection with cells”, and “co-infection” settings (*p* > 0.05) (Figure 6B). However, under the “post-infection” condition, the EV-A71 RNA level was significantly decreased by cordycepin compared to the vehicle control, supporting that the anti-EV-A71 effect of cordycepin occurs after the virus enters the cell (*p* < 0.05) (Figure 6B). In addition, treatment with 100 μM cordycepin after infection significantly reduced the intracellular levels of EV-A71 VP1 protein compared to the vehicle control (Figure 6C). In the quantified data, the ratio of EV-A71 VP1 protein to DAPI fluorescence intensity was significantly decreased in EV-A71-infected Vero cells treated with 100 μM cordycepin, as compared to the nontreated control (*p* < 0.05) (Figure 6D).

### 3.6. Exogenous Adenosine Reduces the Anti-EV-A71 Effect of Cordycepin

**Figure 7:**
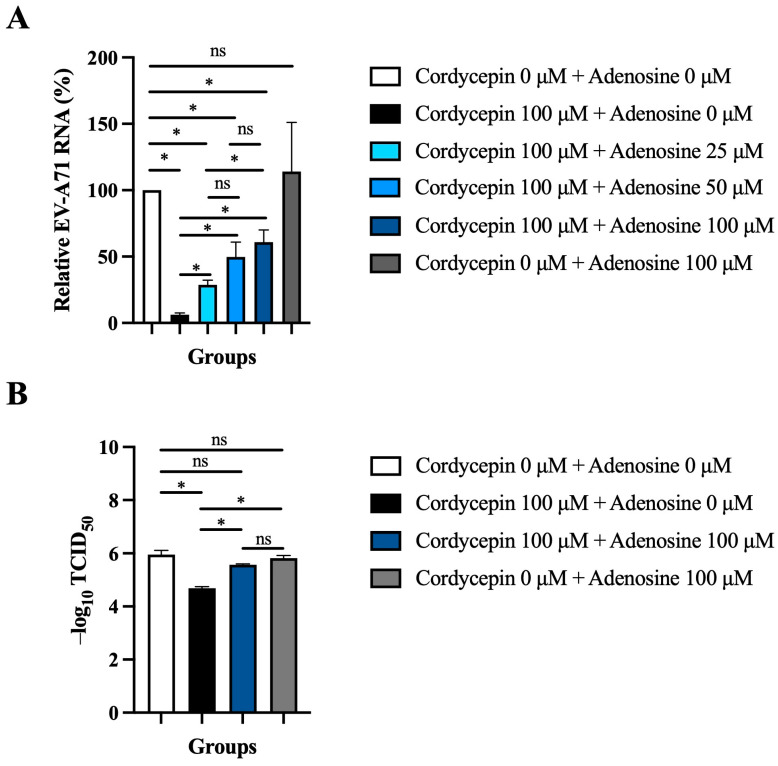
Exogenous adenosine reduces the anti-EV-A71 effect of cordycepin. (**A**) Vero cells were infected by EV-A71/MP4 at an MOI of 0.1, and then treated with or without 100 μM cordycepin and increasing doses of adenosine after 1-h virus adsorption. Cultures (cells with supernatants) were harvested for RNA isolation at 48 h post-infection, followed by conversion to cDNA. Viral RNA levels were quantified by real-time RT-PCR with EV-A71-specific primer pairs and normalized by the mRNA levels of GAPDH. Data are presented as mean ± SEM of three independent experiments in percentages relative to the control group (cordycepin 0 μM + adenosine 0 μM). (**B**) Vero cells were infected with a serial dilution of EV-A71/MP4 in a 96-well plate, and then treated with or without 100 μM cordycepin and adenosine (0 or 100 μM) after 1-h virus adsorption. After 72 h, CPE was examined and TCID_50_ values were calculated using the Reed and Muench formula. Data are presented as mean ± SEM of three independent experiments. * *p* < 0.05 represents significant statistical difference. ns, no significant difference.

Since cordycepin is an analog of adenosine, it is possible that the anti-EV-A71 effect of cordycepin acts through the adenosine pathway. To test this hypothesis, the adenosine competition assay was performed. The inhibition of 100 μM cordycepin on EV-A71 RNA levels was gradually weakened with the increase in adenosine concentration in a dose-dependent manner (*p* < 0.05, cordycepin 100 μM + adenosine 0 μM vs. cordycepin 100 μM + adenosine 25/50/100 μM) (Figure 7A). Furthermore, the addition of 100 μM adenosine significantly reduced the TCID_50_ value (*p* < 0.05) that had been increased by 100 μM cordycepin treatment in Vero cells (Figure 7B).

## 4. Discussion

Cordycepin, also known as 3′-deoxy-adenosine, is the main biologically active ingredient in the natural source “*Cordyceps sinensis*” [45]. *Cordyceps sinensis* is a medicinal material frequently used in traditional Chinese medicine, and its relative safety and good tolerability are considered advantages for clinical use. In this study, we reveal in vitro evidence that cordycepin inhibits EV-A71 replication, and protects host cells from EV-A71-induced cytotoxicity. Its anti-EV-A71 pharmacological effect is mainly achieved through the adenosine action pathway, and it exerts an inhibitory effect at the post-infection stage of the viral infection, resulting in reduced virus titer and viral RNA levels, while the TCID_50_ value increases. In addition, the anti-EV-A71 activity of cordycepin was verified in the Vero cell model, and the inhibitory effect can also be extended to Caco-2 intestinal cells. These results suggest that cordycepin may inhibit the early replication of EV-A71 after infecting the host, and control the progression of viral infection in vivo. Our findings highlight the potential of cordycepin as a therapeutic agent for EV-A71 infection.

In recent years, some research evidence supports certain nucleoside analogs, such as forodesine [46], riboprine [46], ensitrelvir [47], didanosine [48], adefovir [49], and cordycepin [37], as having excellent antiviral capabilities, especially against SARS-CoV-2. These nucleoside analogs may inhibit viral genome synthesis and replication by affecting viral polymerase or other enzymes essential for replicating viral genetic materials [46,47,48]. In the present study, we observed that cordycepin reduced the level of viral RNA in EV-A71-infected cells (Figure 3). However, whether cordycepin can inhibit EV-A71 replication by affecting EV-A71 viral proteins, such as 3D polymerase, still requires further research to clarify.

The life cycle of EV-A71 is composed of a series of consecutive events, including virus–receptor binding, receptor-mediated endocytosis, uncoating, viral protein synthesis, RNA genome synthesis, assembly, virus maturation, and release from the host cell. Candidate drugs that inhibit any step in the EV-A71 life cycle may reduce virus production [2]. The results of Figure 2, Figure 3, Figure 4 and Figure 5 demonstrate the impact of the long-term presence of cordycepin on the entire process of the EV-A71 life cycle, thereby inhibiting virus production. To understand the specific stages at which cordycepin exerts its anti-EV-A71 effects, we designed the “Time addition assay” to dissect the EV-A71 life cycle and test the stage of cordycepin action (Figure 6A). According to the results in Figure 6B, the inhibitory effect of cordycepin on EV-A71 occurs when the virus enters the host cell, and possible affected steps including uncoating, viral protein synthesis, RNA genome synthesis, assembly, maturation, and release from the host cell. However, cordycepin has no obvious inhibitory effect on virus–receptor binding, receptor-mediated endocytosis, and direct toxicity to the virus. As for which life cycle step cordycepin inhibits EV-A71 after entering cells and what the molecular mechanism is, these issues can be further explored in the future.

Adenosine receptors are G protein-coupled receptors that include four subtypes, A1, A2A, A2B, and A3, which are widely expressed in a variety of cell types [50]. Cordycepin has high affinity for all adenosine receptor subtypes, and can regulate different physiological effects through binding to different receptor subtypes [51,52]. The results in Figure 7 support the anti-EV-A71 activity of cordycepin mainly being achieved through its binding to adenosine receptors, which may then further regulate cell signaling to build cellular defense against the virus. A previous report has pointed out that cordycepin can bind to the adenosine A2A receptor subtype, subsequently promoting the activation of the downstream protein kinase A (PKA)-related pathway, and stimulating the production of cytokines to regulate immune responses [50]. In some cases, the interaction of cordycepin with adenosine receptors, especially the A2A and A3 subtypes, can lead to the suppression of inflammatory responses, which may retard the immunopathological processes caused by EV-A71 infection [50]. It has also been reported that activation of the adenosine A3 receptor-related pathway leads to inhibition of adenosine cyclase, thereby reducing the level of cyclic adenosine monophosphate (cAMP) [53]. The low intracellular cAMP environment may lead to blockage of viral protein synthesis and viral replication. In the future, it is necessary to further determine the affinity of cordycepin to different adenosine receptor subtypes when cells are infected with EV-A71, as well as the impact of adenosine pathway activation on downstream signaling pathways, in order to elucidate the detailed molecular mechanism of cordycepin’s anti-EV-A71 effect.

In addition to achieving the anti-EV-A71 effect by activating adenosine receptor-related pathways, cordycepin also has a variety of biological functions that may affect viral replication. The data in Figure 7A show that 100 μM adenosine cannot completely abolish the 100 μM cordycepin-mediated inhibition of EV-A71 RNA production, suggesting that other auxiliary mechanisms may be involved. It has been reported that that cordycepin can cause down-regulation of the appearance of mRNA in cells by inhibiting polyadenylate synthesis [54], indicating that cordycepin administration may also cause genetic instability of EV-A71. In addition, the functional RNAs of the EV-A71 genome are highly 3′-polysdenylated [6]. If cordycepin can increase the instability of viral RNA by inhibiting the polyadenylation process, it will inevitably further affect viral proteins translation and viral genome replication, ultimately reducing the viral load in the host cell [40]. Furthermore, our previous study found that the length of the poly(A) tract plays an important role in the variability of the genetic pool of the tick-borne encephalitis virus [43]. Viruses with short poly(A) tracts are more genetically stable than viruses with long poly(A) tracts. Viral quasispecies with high genomic diversity have been shown to have better adaptability to changing host environments [55]. Therefore, the anti-EV-A71 effect of cordycepin may also be related to regulating the length of the virus poly(A) tract. On the other hand, both cordycepin administration and EV-A71 infection are able to modulate host cell apoptosis, autophagy, and reactive oxygen species (ROS) induction [35,56,57]. Whether such interactions eventually cause the suppression of EV-A71 replication needs further clarification. Moreover, EV-A71 is sensitive to the treatment of type I interferon (IFN) [22,58]. Cordycepin is structurally and functionally similar to an antiviral complex, (2′-5′) oligoadenylate (2–5A), induced by type I IFN [59,60]. Whether cordycepin can inhibit EV-A71 replication through 2–5A-related antiviral mechanisms, or feedback-regulate other antiviral responses mediated by type I IFN, can also be studied in the future. Importantly, because cordycepin has multiple pharmacological effects, the safe dosage of the drug should be carefully evaluated before clinical application in the future, in order to reduce the possibility of side effects.

Panya et al. have shown that cordycepin can inhibit dengue virus replication and viral RNA synthesis, and those inhibitory effects only occur at the post-infection stage [37]. These research results support our findings on EV-A71, and suggest that the antiviral effects of cordycepin may have broad-covering effects on different viruses through a similar mechanism. In terms of antiviral efficiency, the inhibitory effects of 100 μM cordycepin on dengue virus and EV-A71 titers were two log-fold and one log-fold decreases at 48 h post-infection, respectively. In terms of antiviral timeliness, 100 μM cordycepin inhibited dengue viral RNA synthesis and viral titers at 8 and 24 h after infection, respectively, while for EV-A71, significant inhibitory effects occurred at 48 h post-infection. In comparison, although the antiviral effect of cordycepin against EV-A71 is slightly weaker and slower than that against dengue virus, our data support cordycepin being effective against EV-A71 infection. Therefore, possible future clinical applications may involve its use in the treatment of mild EV-A71 infections. In mild cases, the patient’s immune system may have been predisposed to fight the virus. In this situation, treatment with cordycepin may be able to further synergistically slow down the progression of EV-A71 infection, and prevent the virus from invading the CNS and causing severe disease. In addition, the combination treatment of cordycepin with other drug candidates, such as type I IFNs, milrinone, IVIG, ribavirin, and pleconaril, may improve the therapeutic effect and antiviral immediacy to overcome the timeliness issue of medical treatment.

In 1976, Panicli and Nair’s research revealed for the first time that cordycepin can inhibit the replication of picornavirus by affecting the synthesis of polio virus RNA; this phenomenon can be reversed by polyadenylic acid, indicating that virus-specific RNA synthesis is the target, and competition with adenosine is the mechanism of cordycepin action [39]. Afterwards, they further presented evidence that the inhibition of polio virus replication is due to direct interference by 3′-deoxyadenosine triphosphate (3′-dATP) with viral RNA synthesis [61]. These results support our findings that the effect of cordycepin against EV-A71 is achieved through the adenosine action pathway, which also implies that the anti-EV-A71 effect of cordycepin may be extended to different picornaviruses, including different enteroviruses and human parechoviruses. Thus far, the scientific literature on cordycepin against picornavirus is quite rare. More research is needed in the future to evaluate the effect of cordycepin on individual viruses. Our current findings can just supplement the knowledge in this field.

There are two limitations of this study. Firstly, we only evaluated the antiviral effects of cordycepin using two strains of EV-A71. Future research could include more EV-A71 strains or other picornaviruses for efficacy evaluation, such as coxsackieviruses and echoviruses. Secondly, while the current study provides rigorous in vitro evidence of cordycepin’s anti-EV-A71 activity, there is still a lack of in vivo data to support these findings. However, a previous study has reported the inhibitory effect of cordycepin on the replication of type-c RNA tumor viruses in mice [62], indicating that cordycepin could exert antiviral bioactivity in vivo. In the future, further animal experiments are still needed to evaluate the safety, effectiveness, bioavailability, administration route and dosage, pharmacokinetics, and other biological indicators in vivo to maximize the therapeutic potential of cordycepin.

## 5. Conclusions

**Figure 8:**
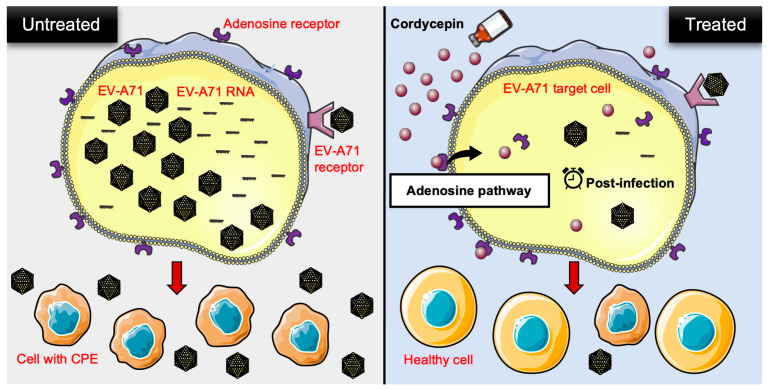
Schematic representation of cordycepin inhibiting EV-A71 replication and protecting host cells from virus-induced cytotoxicity through an adenosine action pathway. In the present study, the authors provided in vitro evidence to demonstrate that cordycepin, a constituent isolated from the mycelia of *Cordyceps sinensis*, not only reduces the viral load, but also lowers viral RNA levels in EV-A71-infected cells. On the other hand, EV-A71-mediated cytotoxicity is decreased after cordycepin treatment in host cells. This anti-EV-A71 effect may operate through the adenosine pathway of action during the post-infection stage. CPE: cytopathic effect.

Our study reveals a novel finding that cordycepin exhibits anti-EV-A71 activity. It not only inhibits EV-A71 replication, but also protects host cells from EV-A71-induced cytotoxicity. The inhibitory effect can be reproduced in different EV-A71 permissive cells, and in response to different EV-A71 strains. Cordycepin achieves its anti-EV-A71 effect after the virus enters the cell, and mainly acts through the adenosine pathway (Figure 8). Our findings suggest that cordycepin has potential as a drug candidate for treating EV-A71 infection. The future applications of cordycepin may involve its use in treating mild EV-A71 infections, such as HFMD or herpangina, to alleviate symptoms, shorten the clinical course, and reduce the risk of severe complications.

## Figures and Tables

**Table 1:** List of real-time RT-PCR primers.

Target Gene	Forward Primer	Reverse Primer
Simian GADPH	GGGAGCCAAAAGGGTCATCA	CGTGGACTGTGGTCATGAGT
EV-A71/MP4	GAGAGTTCTATAGGGGACAGT	AGCTGTGCTATGTGAATTAAGAA
EV-A71/Tainan/4643/98	GAGAGTTCTATAGGGGACAGT	AGCTGTACTATGTGAATTAAGAA

## Data Availability

Data are contained within the article.

## Supplementary Materials

The following supporting information can be downloaded at: https://www.mdpi.com/article/10.3390/v16030352/s1, Figure S1: CCK-8 viability test-related O.D. values of mock- or EV-A71-infected Vero cells in the absence or presence of cordycepin; Figure S2: Toxicological test of cordycepin in Caco-2 cells.
