# Supplementary material for: Cordycepin Inhibits Enterovirus A71 Replication and Protects Host Cell from Virus-Induced Cytotoxicity through Adenosine Action Pathway
Source: Viruses. 2024 Feb 24;16(3):352. doi: 10.3390/v16030352 (PMC10974990; doi:10.3390/v16030352)
Supplement: Supplementary file 1 [file viruses-16-00352-s001.zip › viruses-2863784-supplementary.pdf]

Supplementary Materials

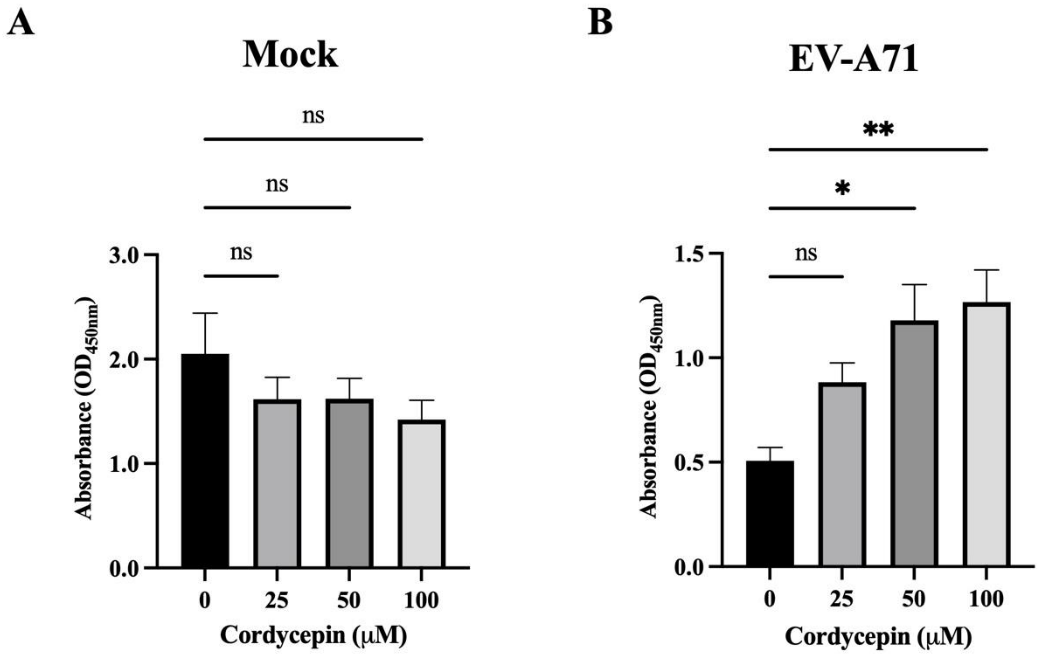

Figure S1. CCK-8 viability test-related O.D. values of mock- or EV-A71-infected Vero cells in the absence or presence of cordycepin. (A) Vehicle or (B) EV-A71/MP4 viruses were added to Vero cell cultures at a MOI of 0.1 and treated with 0, 25, 50 or 100 μM cordycepin for 96 hours. Cell viabilities were examined by CCK-8 viability test and the related O.D. values were shown. Data are presented as mean ± SEM of three independent experiments. *$p < 0.05$, and **$p < 0.01$ represent significantly statistical difference compared to the untreated control group. ns, no significant difference.

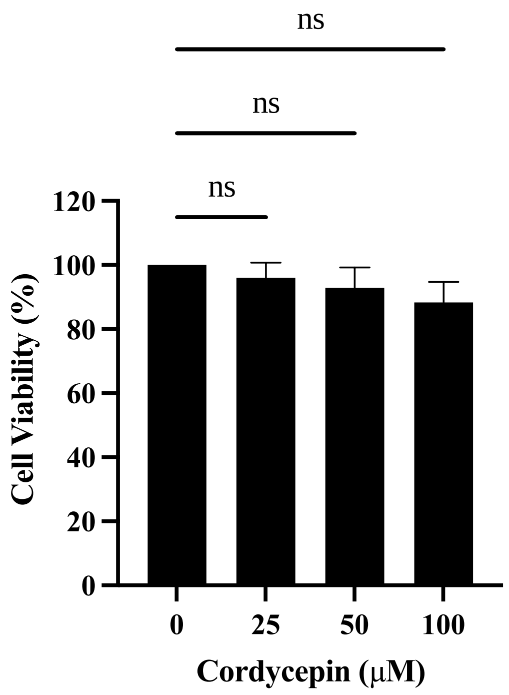

Figure S2. Toxicological test of cordycepin in Caco-2 cells. Caco-2 cells were treated without or with 25, 50, and 100 μM cordycepin in 100 μl of final volume for 72 hours. 3-(4,5-Dimethylthiazol-2-yl)-2,5-diphenyltetra-zolium bromide (MTT) were added with the final concentration of 0.5 mg/ml, and then incubated at 37°C for 4 hours. After incubation, the medium was removed and 100 μl DMSO was added into each well to dissolve the crystals. The O.D. values in each treatment were determined under the absorbance at 570 nm by ELISA reader. Results are presented as mean ± SEM of three independent experiments in percentages of cell viability relative to the untreated control group. ns, no significant difference compared to the untreated control group.
